# Midbrain adaptation may set the stage for the perception of musical beat

**DOI:** 10.1098/rspb.2017.1455

**Published:** 2017-11-08

**Authors:** Vani G. Rajendran, Nicol S. Harper, Jose A. Garcia-Lazaro, Nicholas A. Lesica, Jan W. H. Schnupp

**Affiliations:** 1 Auditory Neuroscience Group, Department of Physiology, Anatomy, and Genetics, University of Oxford, Oxford, UK; 2 UCL Ear Institute, 332 Grays Inn Rd, Kings Cross, London WC1X 8EE, UK; 3 Department of Biomedical Sciences, City University of Hong Kong, 1/F, Block 1, To Yuen Building, 31 To Yuen Street, Hong Kong

**Keywords:** beat, rhythm, electrophysiology, psychophysics, temporal processing, perception

## Abstract

The ability to spontaneously feel a beat in music is a phenomenon widely believed to be unique to humans. Though beat perception involves the coordinated engagement of sensory, motor and cognitive processes in humans, the contribution of low-level auditory processing to the activation of these networks in a beat-specific manner is poorly understood. Here, we present evidence from a rodent model that midbrain preprocessing of sounds may already be shaping where the beat is ultimately felt. For the tested set of musical rhythms, on-beat sounds on average evoked higher firing rates than off-beat sounds, and this difference was a defining feature of the set of beat interpretations most commonly perceived by human listeners over others. Basic firing rate adaptation provided a sufficient explanation for these results. Our findings suggest that midbrain adaptation, by encoding the temporal context of sounds, creates points of neural emphasis that may influence the perceptual emergence of a beat.

## 1. Introduction

When listening to a rhythmic sound such as music, one can often find and tap along with a steady (isochronous) beat. In principle, many possible interpretations of beat could exist, but in practice, only very few of them tend to be chosen by listeners. What could be the neurophysiological determinant for where in a rhythmic sound the beat is felt?

At the highest level, human studies have revealed beat-specific entrainment of cortical oscillations [1–5]. A promising candidate mechanism for the entrainment of these oscillations may be the cortico-basal ganglia–thalamocortical loop [6], of which the basal ganglia are thought to be particularly important for beat perception [7–9] and time perception [10,11]. If the dynamics of these circuits serve to select an interpretation of beat out of the many theoretically possible ones, then beat-relevant precursors may already be present as a result of low-level auditory processing. However, the influence of low-level auditory processing on-beat perception has not yet been characterized. We hypothesized that adaptive processes in the brainstem, which are common across mammalian species [12–16] regardless of whether they can synchronize their movements to a rhythmic stimulus [17], may strongly influence which beat interpretations are chosen over others. We tested this hypothesis by investigating the correspondence between brainstem processing in a rodent model and beat perception in humans.

Seven rhythmic patterns [18] constructed from identical broadband noise bursts were played to anaesthetized gerbils while recording from the central nucleus of the inferior colliculus (IC), the major midbrain relay through which ascending information from the auditory nerve passes on its way to the cortex. The same rhythms were also played to human listeners who simply tapped along to the beat they perceived. While the influential study by Nozaradan *et al*. [18] provided the inspiration for this work, the frequency domain method used in that study does not yield a dependable measure of beat entrainment (see electronic supplementary material, figure S4). Here, we present a novel time domain analysis that avoids ambiguities in the interpretation of frequency domain-based analyses [19]. On average, neural activity recorded from the midbrain was stronger on the beat than off the beat, and this asymmetry was a defining feature of the beat interpretations chosen out of all possible ones. Furthermore, adaptation provided a parsimonious account for our results. Together, these findings strongly support the possibility that midbrain adaptation may already be shaping where the beat is ultimately perceived in musical rhythms.

## 2. Results

### (a) On-beat sounds evoke stronger neural responses on average than off-beat sounds

We hypothesized that low-level stimulus processing might already create a neural ‘emphasis’ that accompanies the perceptual emphasis felt on the beat, and that this emphasis might be observable as higher firing rates on the beat than off the beat.

**Figure 1.**
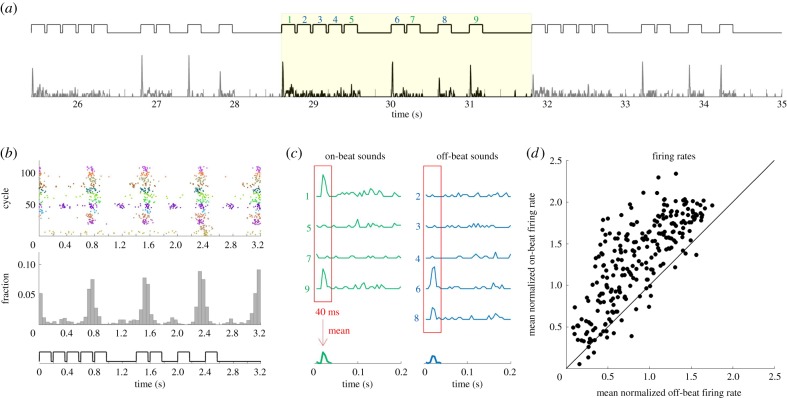
On-beat responses are on average stronger than off-beat responses. (*a*) An example unit's normalized peri-stimulus time histogram (PSTH) to stimulus pattern P2 at 1 × tempo. The last three complete cycles of this pattern are shown together with the stimulus trace above. The coloured numbers indicate noise bursts, green being on-beat and blue being off-beat. Normalization was done by dividing each unit's firing rate concatenated across all seven stimuli by its standard deviation. (*b*) Raster-style plot showing tap times of human listeners across trials and cycles of this stimulus. Each dot marks the timing of a tap, each row of dots shows tap responses for a single cycle of the stimulus pattern and different colour dots distinguish the tap responses from different subjects. Beneath is a histogram of tap times pooled across subjects, cycles and trials for this stimulus. A clear, regular tapping pattern is present, which indicates a strong consensus among listeners to hear four beats in this 16-event sequence, timed on the 1st, 5th, 9th and 13th interval. (*c*) Normalized firing rate in response to on-beat (left, in green) and off-beat (right, in blue) noise bursts for the highlighted cycle shown in panel *a*. To quantify a unit's mean on- and off-beat response, the firing rate over the first 40 ms of each sound-evoked response to on-beat and off-beat sounds (red boxes) across all 33 s of all stimuli was averaged (the averages based on just the highlighted cycles are shown for demonstration purposes), and the mean of the result was taken. (*d*) Scatter plot showing the mean on-beat response for each unit as a function of that unit's mean off-beat response. Most points fall well above the main diagonal, indicating that, on average, on-beat sounds evoked stronger responses than off-beat sounds.

Neural activity was recorded extracellularly from 29 single units and 220 multi-units originating from 149 recording sites in the central nucleus of the IC of four gerbils in response to seven rhythmic sound patterns. The chosen rhythmic sound patterns were taken from a previous human electroencephalography (EEG) study [18] and are described in detail in the Material and methods. Each sound pattern consisted of a repeated sequence of 12 or 16 equal-duration ‘events’ separated by brief silent gaps, where events were either silent intervals or identical broadband noise bursts. Beat locations for each rhythm were determined based on the most common tapping pattern across 13 human listeners (figure 1*b*; see electronic supplementary material, figure S1 for tapping data for all stimuli). Noise bursts were classified as either being ‘on-beat’ if taps were aimed at their onset, or ‘off-beat’ otherwise. The mean firing rate during the first 40 ms of all on-beat and off-beat noise bursts was calculated for each unit (figure 1*c*). A time window of 40 ms was chosen because it was the longest time window that allowed comparison across all seven stimuli because it corresponded to the duration of the noise burst in the fastest pattern. Figure 1*d* illustrates each unit's average on-beat and off-beat response across all sound events and all rhythmic patterns (see electronic supplementary material, figure S2 for on-beat versus off-beat responses for each stimulus individually). On average, neural responses were significantly stronger to on-beat sounds than to off-beat sounds, despite all sound events being acoustically identical noise bursts (figure 1*d*, *p* < 10^−6^, Wilcoxon paired signed-rank test, *n* = 248 units).

### (b) A large on-beat neural emphasis is a defining feature of the perceived beat

Next, we explored whether a large neural emphasis on the beat might explain why some beat structures were more commonly perceived than other possible ones. Our hypothesis here is that sound events that evoke a particularly strong neural response are more likely to be perceived as being on-beat, which would support the possibility that systematic differences in evoked response strength at low levels of the auditory pathway might predetermine both the grouping and phase of the beat interpretation that is ultimately selected at higher levels.

A beat structure as we define it consists of a beat period and a beat position. For a given rhythmic pattern, the beat period is determined by the integer number of events (noise bursts or silent intervals) that listeners grouped together while tapping. The beat position refers to the temporal frame or ‘phase’ of the tap intervals, and is set by the time points that the listeners report as on-beat with their taps. Beat periods were considered ‘hypothetically possible’ if they divided up the rhythmic pattern into an integer number of equal length intervals. Beat periods that were not an integer fraction of the number of events in the pattern would have put the beat on different places in successive repetitions of the pattern, and none of our subjects exhibited such a ‘beat precession.’ Thus, for rhythms P1 and P3, both 12-event patterns, possible beat periods consisted of tapping a beat once every 2, 3, 4, 6 or 12 events, while for the 16-event patterns, possible beat periods would be 2, 4, 8 or 16 events long. Note that we do not consider a beat period of 1, where all events would be on-beat with none off the beat. Possible beat phases refer to the *N* possible positions at which the beat could start for a beat period that contains *N* events. Twelve-event patterns therefore had 2 + 3 + 4 + 6 + 12 = 27 possible beat structures, while the 16-event pattern 2 + 4 + 8 + 16 = 30 possible beat structures.

Population neural activity was calculated as the average firing rate across all single and multi-units. We classified the mean event-evoked population firing rate during the first 40 ms of each event (noise burst or silence) as being on-beat or off-beat, where ‘on-beat’ and ‘off-beat’ were defined by each beat period and beat position combination for each possible beat structure. For comparison, the average sound content at on-beat and off-beat positions was calculated for each beat structure, counting a sound event as 1 and a silent interval as 0. The on-beat emphasis for sound content and population neural activity was calculated as the difference between its mean on-beat and off-beat values (*on–off*).

**Figure 2.**
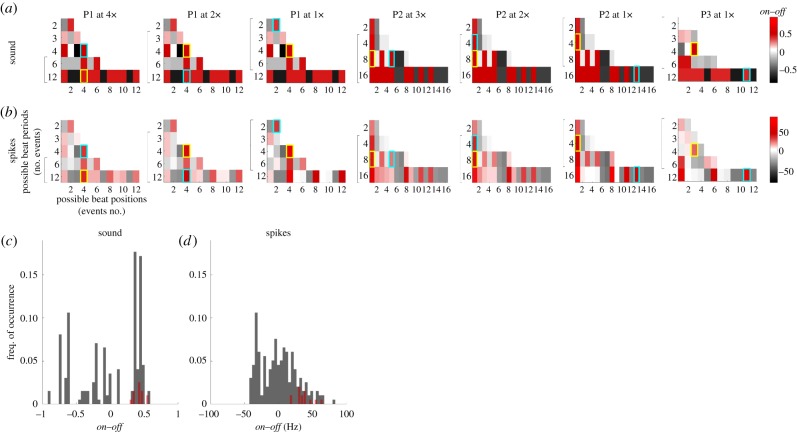
Exploration of *on–off* for all possible combinations of beat periods and beat positions. (*a*) Heat maps represent *on–off* for all possible combinations of beat grouping (*y*-axis) and beat position (*x*-axis) for each of the seven rhythmic sound patterns tested. Yellow and cyan boxes mark the most and second most common beat interpretations reported by human listeners, respectively. Brackets indicate those groupings whose theoretical tapping frequencies are in the range 0.5–4 Hz, the range within which beat is typically perceived [20]. (*b*) Same as panel *a*, but based on population firing rates. (*c*) All hypothetical on–off values for sound (grey) and the two most commonly perceived beat structures (red), pooled across the seven stimuli. (*d*) Same as panel *c*, but for population firing rates.

Figure 2*a,b* shows the *on–off* values for all possible beat structures in the seven stimulus patterns tested. Yellow and cyan boxes mark the most and second most commonly perceived beat structures, respectively. All *on–off* values for sound and population neural activity, pooled across the seven stimuli, are shown in the histograms in figure 2*c* and 2*d*, respectively. The beat structures preferred by our listeners, shown in red, have significantly larger *on–off* values than the pool of all possible beat structures, shown in grey, for both sound and neural activity (firing rates: *p* < 10^−6^; sound: *p* < 10^−4^, Wilcoxon rank-sum test, *N* = 198 possible beat structures and 14 perceived beat structures).

It follows from a preference for beat structures with high on-beat sound content that on-beat positions would evoke relatively large on-beat neural responses. However, a consequence of neural processing is that relatively fewer possible beat structures overall resulted in high *on–off* values (figure 2*c,d*). Quantitatively, the distribution over all *on–off* values for firing rates (figure 2*d*) showed higher skewness, or a longer right tail, than the distribution of *on–off* for sound content (figure 2*c*; *p* < 0.01, paired sign test, *N* = 7 stimulus patterns).

Taken together, these findings would suggest that, out of the pool of all possible beat structures, those that are perceived by listeners tend to be those with a relatively large on-beat neural emphasis and relatively high underlying on-beat sound content, and that midbrain processing of sound further restricts the set of possible beat structures that later stages of the nervous system may select. Though commonly chosen beat structures show high *on–off* values for both sound content and population neural activity, it is the neural activity that better distinguishes from all candidate beat structures those that were actually perceived by listeners.

### (c) Strength of on-beat neural emphasis varies systematically with the profile of each recorded inferior colliculus unit's firing pattern

**Figure 3.**
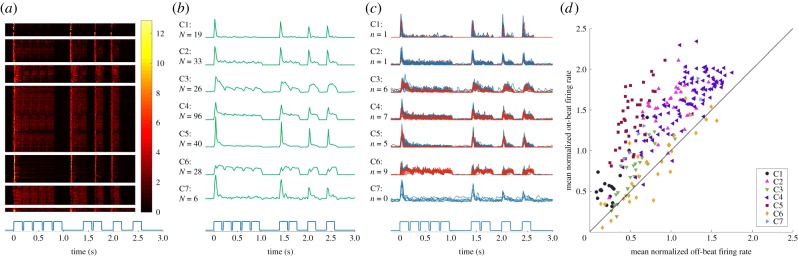
Clustering analysis. (*a*) Heat map illustrating each unit's normalized firing rate in response to one cycle of pattern P2 at 1× tempo. Units are grouped by cluster. The stimulus pattern is plotted beneath each panel for reference. (*b*) Each cluster's mean PSTH, with the total number of units in each cluster (*N*) displayed to the left of each trace. (*c*) Mean PSTH traces of multi-units (blue) and single units (red) in each cluster. The number of single units (*n*) is displayed to the left. (*d*) Same as figure 1*d*, but with units labelled by the cluster to which they belong.

A key advantage of extracellular recordings over non-invasive imaging techniques is the temporal and spatial resolution to observe response dynamics of single cells (single units) and small groups of cells (multi-units). Response patterns in the IC are known to be highly diverse, and to investigate the contribution of neurons with different response properties on the observed on-beat emphasis at the neural population level, we first performed hierarchical clustering on normalized single-unit and multi-unit firing rates in response to all seven stimulus patterns concatenated across time (see Material and methods). This resulted in eight clusters containing our 249 single and multi-units (figure 3*b*), of which one cluster was excluded because it contained only one unit. Previous studies in IC [21,22] suggest that single-unit and multi-unit responses are comparable, and this would appear to be the case for our data too (figure 3*c*). The seven clusters identified are not meant to provide a definitive or exhaustive categorization of the response types that exist within IC, but simply provide a principled set of response profiles that are representative of our data and show characteristics of ‘onset,’ ‘on-sustained’ and ‘sustained’ firing patterns previously identified in the IC [23]. ‘Onset’ (C1, C5, C7), ‘on-sustained’ (C2–C4) and ‘sustained’ (C6)-type units roughly account for 26%, 63% and 11% of our sample, respectively.

To test whether the on-beat neural emphasis varied by response shape, the normalized on-beat and off-beat sound-evoked responses were re-examined from figure 1*d*. Labelling units by cluster reveals that ‘onset’-type units (C1, C5, C7) appear furthest from the diagonal and therefore show the strongest emphasis to on-beat sounds (figure 3*d*). ‘Sustained’-type units (C6) appear near the diagonal, and ‘on-sustained’-type units (C2–C4) fall in between.

### (d) Adaptation may explain why neural responses are stronger on the beat

We hypothesized that a simple explanation for the evident beat processing occurring in the midbrain could be firing rate adaptation, which is the tendency for neural firing rates to decrease to a stimulus if it is prolonged or repeated at high rates. In the context of our patterns, adaptation would result in relatively weaker responses to sounds that are preceded by other sounds in the recent past, and in relatively stronger responses to sounds that are preceded by long periods of silence. This pattern is observable qualitatively (figure 3), so we asked whether firing rate adaptation could explain what we have so far quantified as the contribution of midbrain activity towards beat processing.

**Figure 4.**
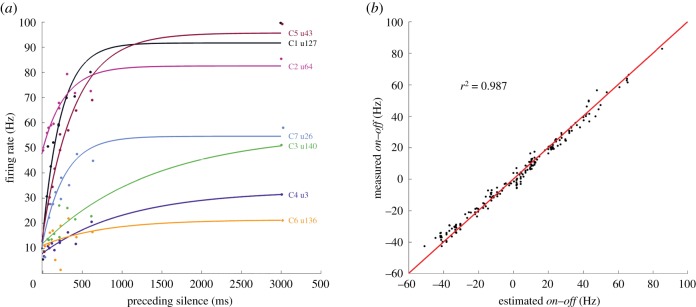
Firing rates estimated from exponential fits closely match measured *on–off* responses. (*a*) The average firing rate evoked by noise bursts preceded by varying amounts of silence is used to fit an exponential to each unit. Data and exponential fits for an example unit from each cluster are shown. (*b*) Scatterplot shows a high correspondence between estimated *on–off* values based on exponential fits (*x*-axis) and the real data (*y*-axis).

Adaptation was quantified for each unit by fitting an exponential function to its sound-evoked firing rate as a function of the amount of silence immediately preceding the sounds. Silent intervals ranged from the 10 ms between consecutive noise bursts to the 3 s that separated each 33 s rhythmic pattern from the next. Data and exponential fits from an example unit from each cluster are shown in figure 4*a*. When the population firing rate was calculated using each unit's exponential fit rather than the real data, the resulting *on–off* values for all candidate beat structures showed a very high correlation with the real data (figure 4*b*), with an *R*^2^ value of 0.987 (see electronic supplementary material, figure S2 for *on–off* heat maps and histograms based on model estimates of the firing rate). Adaptation is therefore likely to be a parsimonious account for why neural responses in the gerbil IC to otherwise identical sounds differ depending on the temporal context, and our results (figures 1 and 2) suggest that these differences may be of direct relevance for beat perception.

## 3. Discussion

By using an approach that combines rodent electrophysiology and human psychoacoustics, we present evidence that low-level processing of rhythmic sounds may exert a more direct influence on beat perception than previously appreciated. Across the set of rhythmic sound patterns tested, the perceptual emphasis felt as a beat was accompanied by a subcortical neural emphasis. An evaluation of all hypothetically possible interpretations of beat for each rhythmic pattern revealed that a large on-beat neural emphasis may be a defining feature of the beat structures actually perceived by listeners, perhaps explaining the tendency for listeners to typically agree on their interpretation of beat for a given rhythm. A diverse set of firing patterns contribute to the on-beat neural emphasis observed at the population level, and we showed that ‘onset’-type cells in the IC show the largest on-beat neural emphasis compared to other cell types. These results could be sufficiently explained through a simple exponential fit that models adaptation.

An asymmetry in response strength to acoustically identical sounds comprising a rhythmic pattern has been previously reported in humans based on cortical EEG [24–26]. The asymmetry in responses observed in the human studies, however, reflected subjective accenting, which is a cognitive, attention-driven process. By contrast, our results, despite showing a similar emphasis on some sounds over others despite all sounds being identical, are not a reflection of high-level or cognitive processes because they can be well described by a simple adaptation model. This strongly suggests that some preprocessing of sound that is relevant to beat perception is already occurring in the brainstem. Adaptation time constants in the IC are robust to a number of stimulus manipulations including duty cycle [13]; it would be interesting to find stimulus manipulations that do alter adaptation and investigate whether such manipulations also alter beat perception in a consistent manner. Adaptation has been characterized throughout the auditory system across a wide range of mammalian species, including humans (see [27] for a review). This cross-species generality strongly suggests that the adaptive mechanisms described in this study are also present in humans during beat perception. Though the response patterns we see may be better described as a form of onset detection rather than beat-specific processing, the (average) physiology need not have corresponded with the chosen beat, and adaptation need not have described the physiology. Our results therefore implicate subcortical adaptation as a relevant stage in beat processing, which suggests that beat perception may, to an extent, be an emergent property of auditory processing that is not entirely culturally dependent.

A natural extension of this hypothesis is that though rodents and other non-human species rarely exhibit the ability to synchronize their movements to rhythmic stimuli with the consistency or precision of that of humans [9,17,28], they could nonetheless be able to perceive musical beat. The gradual audiomotor evolution hypothesis [29] is consistent with this idea, suggesting that humans' superior movement-synchronization ability may be due to stronger coupling between auditory and motor areas in humans than in other animals. However, the idea that non-human animals can perceive beat remains to be tested experimentally, perhaps through tasks where animals are asked to discriminate, rather than synchronize their movements to, different rhythmic sound sequences.

Our finding that bottom-up physiology may constrain the set of beat interpretations that can be perceived must be considered in the context of other well-known constraints on-beat perception, including the 0.5–4 Hz frequency range within which a beat is typically perceived [30, p. 28], and a general preference for binary (e.g. 2, 4) groupings over ternary (e.g. 3, 6) or other groupings [30, p. 44; 31]. Furthermore, the perceived beat and its neural signatures can be modulated at will by top-down mental imagery of beat structure [3,32,33]. In the light of these higher-level considerations, we suggest that the perceived beat may ultimately depend on both a sound's adapted representation in the brainstem, and the set of metrical templates that are common to the listener, with our findings providing evidence of a bias towards configurations that maximize the difference between average on-beat and off-beat responses. Interestingly, this hypothesis parallels the construction of many computational beat-detection algorithms, which typically consist of two stages: a *driving function* and a *periodicity detector* [34,35]. The driving function, analogous to the subcortical representation observed here, is a processed version of the raw audio signal, and a range of beat-detection algorithms employ onset detection to arrive at their driving function. The periodicity detector then extracts periodicities from the driving function and determines the most probably interpretation of the beat. Our results suggest that adaptive phenomena that are already present at early stages of the auditory pathway may play an important role in detecting, or perhaps rather, emphasizing onsets, thereby shaping the ‘driving function’ of the beat detector.

Our work may also relate to other theoretical models of beat perception. The rule-based model of Povel & Essens [36] states that perceptual accents, which are felt for sounds that differ in loudness or in frequency relative to their surroundings, can also arise purely through a temporal context. Specifically, they posit that (i) an isolated sound will be perceived as accented, (ii) the second of two similar sounds played sequentially will be perceived as accented and (iii) the first and last of three or greater similar sounds in a sequence will be perceptually accented. The locations of perceptual accents within a rhythm (which may not be at isochronous intervals) determine the period and phase of the most likely periodic pulse. The adaptation mechanisms we observed here would place a neural emphasis on the first sound of any sequence and would thus not explain these empirical observations, which is a likely indication that adaptation is not the whole story. The empirical observations might reflect an intermediate stage between low-level representation and the fully formed beat percept, and it would be an interesting follow-up to determine why, for example, the second of a group of two sounds is perceived as accented when the first would evoke higher firing rates subcortically. Other influential models suggest the importance of the attentional system [37] and the motor system [38–40]. Neural resonance theory, an influential computational model that consists of a ‘sensory’ and a ‘motor’ layer of nonlinear oscillators whose interactions are modelled as a dynamical system, makes explicit predictions about neural activity and perception [41,42].

This study represents an important first step towards understanding how low-level auditory processing drives beat perception, and opens a number of avenues for future exploration. Most interesting among them would be to trace beat processing through different structures in the brain to determine where and how beat-specific neural activity arises. We suggest that the cortico-basal ganglia–thalamo–cortical loop [6] may be a promising circuit to probe. Given that the IC has direct and indirect projections to the thalamus, we speculate that the large periodic pushes we observed from on-beat positions interspersed with relatively quiescent off-beat intervals due to adaptation could play a critical role in coordinating the activity of the cortico-basal ganglia–thalamo–cortical loop in response to rhythmic sounds. This is consistent with human EEG findings that describe neural entrainment to rhythmic sound sequences in the auditory cortex [32,42,43], but not in the auditory brainstem [44]. The exact relationship between cortical entrainment measured using EEG and spiking responses in the cortex is still a crucial open question. The time domain methods developed here are ideal for cross-species exploration of beat processing in different brain areas and can easily be built upon for more complex stimuli such as music. Note that depending on the temporal and spatial resolution of the recording method used, neural response latencies may first need to be subtracted or deconvolved from the signal. Importantly, the time domain methods developed here also avoid the significant shortcomings of the frequency domain method used in the original Nozaradan *et al*. [18] EEG study (see electronic supplementary material, figure S4).

To conclude, we show that brainstem processing may restrict the range of possible beat interpretations for a given rhythmic sound pattern. Onset-type neural responses are particularly important for this type of processing, and this processing is very well captured by a model based on exponential adaptation. Our results imply that a consequential part of beat perception may not be culturally determined but may be due to simple brainstem processes that are in-born or learnt from the low-level statistical characteristics of sensory input [12,13,15]. This observation is a demonstration of how the nature of high-level brain processes is often biased in its characteristics by ‘primitive,’ low-level features of the nervous system.

## 4. Material and methods

### (a) Sound stimuli

**Figure 5.**
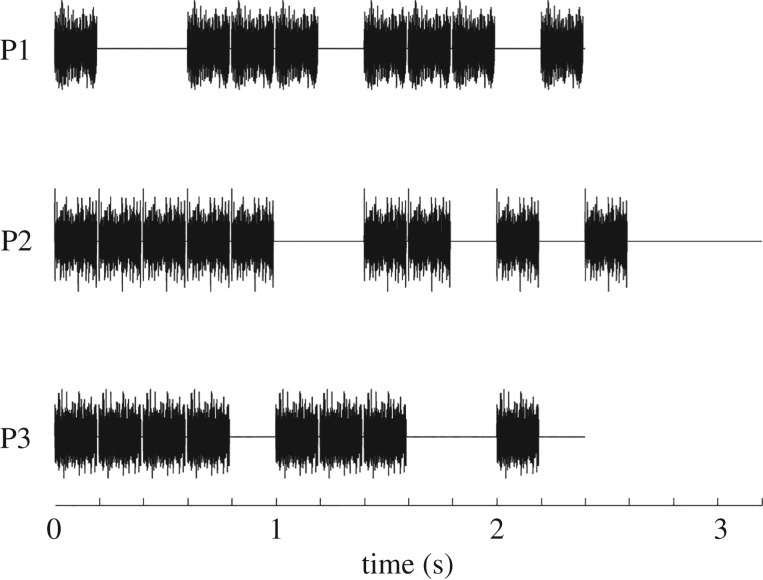
An illustration of the rhythmic patterns P1–P3, from top to bottom. The slowest tempo (1×) is illustrated for each pattern where each event was 200 ms in duration. In the experiment, P1 was played at tempi of 1×, 2× and 4×; P2 at 1×, 2× and 3×; and P3 at 1×.

Seven out of nine sound patterns from Nozaradan *et al.* [18] were recreated, but instead of pure tone bursts we used bursts of frozen pink (1/*f*) noise in order to increase the likelihood of driving neural activity across all recording sites, irrespective of frequency tuning. The patterns used by Nozaradan *et al*. [18] were inspired by the work of Povel & Essens [36] and were designed to preferentially induce a beat percept based on a grouping of four events at the 1 × tempo, with additional beat groupings possible based on subdivision or multiples of the preferred grouping. The seven patterns were constructed from three distinct base patterns (summarized in figure 5), hereafter referred to as P1, P2 and P3, played at different speeds. P1 and P3 consisted of 12 ‘events’ and P2 consisted of 16 ‘events’. At the slowest tempo, events were presented at a rate of 5 Hz, which meant that each event was 200 ms in duration and was either 200 ms of silence or a 190 ms burst of frozen pink noise followed by 10 ms of silence. At this 5 Hz presentation rate, P1 and P3, each consisting of twelve 200 ms events lasted 2.4 s, while P2 lasted 3.2 s. Each pattern was then looped continuously over 33 s.

P1 and P2 were additionally presented at faster event rates, or tempi. In the original study, this was done in order to investigate the effect that the tempo of a sound pattern had specifically on perception and on EEG responses because the shape of the sound envelope spectrum was relatively consistent for the same pattern played at different tempi. P1 was presented at 5 Hz, 10 Hz and 20 Hz, (1 ×, 2 × and 4 × the original tempo), P2 was presented at 5 Hz, 10 Hz and 15 Hz, (1 × , 2 × and 3 × the original tempo) and P3 was presented at 5 Hz only (1 × ). The complete set of seven patterns thus comprised P1 and P2 at three different rates each plus P3 at an event rate of 5 Hz only. At the accelerated tempi, the original 190 ms pink noise token used for the noise events was truncated to 90 ms, 56 ms or 40 ms for the 2 × , 3 × and 4 × conditions, respectively, in each case followed by 10 ms of silence, while the silent intervals were shortened accordingly from 200 ms to 100 ms, 66 ms and 50 ms for the 2 × , 3 × and 4 × conditions, respectively. The tempi and patterns described here are the same as those used in Nozaradan *et al*. [18].

### (b) Electrophysiological recordings

#### (i) Surgical protocol

The recording methods were identical to those used in Schnupp *et al*. [22]. All procedures were approved and licensed by the University College of London, London, UK (UCL) Animal Welfare and Ethical Review Body (AWERB) as well as the UK home office in accordance with governing legislation (ASPA 1986). Four male Mongolian gerbils weighing between 70 and 80 g were anaesthetized with an intraperitoneal injection of 0.65 ml per 100 g body weight of a mixture of five parts of ketamine (100 mg ml^−1^), one part of xylazine (20 mg ml^−1^) and 19 parts of physiological saline. To maintain anaesthesia, the same solution was infused continuously during recording at a rate of approximately 2.1 µl min^−1^. A craniotomy was performed centred on the lambdoid suture and extending 3.5 mm lateral from the midline on the right-hand side. The visual cortex directly dorsal of the IC was aspirated and the sinus was carefully retracted, exposing the IC. Pinnae were removed before the placement of headphones.

Recordings were made using a 64-channel silicon probe (Neuronexus Technologies, Ann Arbor, MI, USA) with 175 µm^2^ recording sites arranged in a square grid pattern at 0.2 mm intervals along eight shanks with eight channels per shank. The probe was inserted into the IC in a medio-lateral orientation for two of the four animals, and in a rostro-caudal orientation for the remaining two animals, in all cases aiming for the central nucleus of the IC.

The seven rhythmic sound stimuli were assembled into a block, with 3 s of silence separating each 33 s stimulus loop from the next. Each block was repeated 10 times at each penetration site. Stimuli were presented binaurally through headphones at 80 dB SPL. Sounds were presented with a sampling rate of 48 828 Hz, and data were acquired at a sampling rate of 24 414 Hz using a TDT system 3 recording set-up (Tucker Davis Technologies).

#### (ii) Data preprocessing

This work made use of the Open Science Data Cloud (OSDC) [45]. Raw voltage traces from the 64-channel recordings were obtained using custom-written Matlab (Mathworks) software. The raw voltage traces were low-pass filtered at 100 Hz and down-sampled to 200 Hz for analysis of local field potentials. Offline spike sorting and clustering was done on the raw data using an automated expectation-maximization algorithm (Spikedetekt/Klustakwik) [46], and clusters were manually sorted using Klustaviewa (Cortical Processing Lab, University College London). Firing rates for multi-units were calculated by binning spike times into 5 ms bins, which resulted in firing rate traces at an effective sampling rate of 200 Hz.

To determine whether multi-unit activity and LFPs were reliably driven by our sound stimuli, a noise power to signal power cut-off of 40 was chosen [47]. Units that failed by this measure of repeatability were excluded from further analysis, leaving 194 recording sites from which local field potentials were analysed, and from which 249 distinct, reliably driven spiking units could be isolated. Of those, 29 were identified as single units and the rest were deemed multi-units. All analysis was performed using custom-written Matlab code.

#### (iii) Clustering

**Figure 6.**
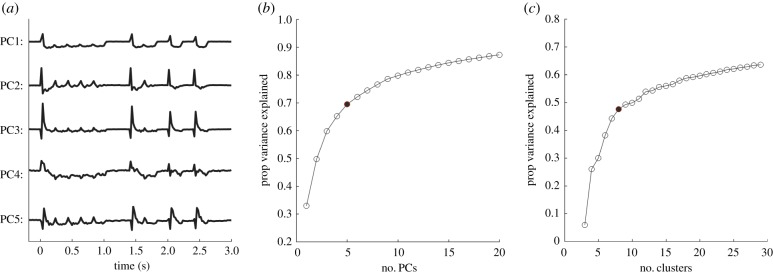
Principal component analysis for hierarchical clustering. (*a*) The first five principal components based on normalized firing rates. (*b*) The elbow method was applied to decide that five principal components would be used for clustering. (*c*) The elbow method applied again to decide that eight clusters adequately explained variance in the data. One of the eight clusters contained only one unit, and this cluster was discarded from further analysis, yielding the seven clusters referred to throughout. (Online version in colour.)

To organize the variety of response patterns observed among IC units into representative groups, we clustered their period peri-stimulus time histograms (PSTHs) using a two-step process consisting of a principal component analysis (PCA) to reduce the dimensionality of the response patterns, followed by standard hierarchical clustering (figure 6). First, the PSTH of each unit in response to pattern P2, binned at 20 ms, was normalized to have ‘unit power’ by dividing the PSTH by the standard deviation across all bins. To reduce the dimensionality of each response down from 160 time bins, the PSTH vectors were ‘centred’ by subtracting their mean. Finally, PCA was performed using the Matlab function *princomp*.

The ‘elbow method’ was applied to determine a cut-off in the number of PCs beyond which the proportion of variance explained began to asymptote (figure 6*b*). Five PCs (out of *N* = 249), which together accounted for 70% of the variance, was chosen as the cut-off. The first five principal component loadings for each unit were then subjected to hierarchical clustering using the Matlab function *cluster* with a Euclidean distance metric. To determine the number of clusters in the data, the percentage of response variability accounted for by optimally splitting the dataset into between three and 30 clusters was calculated. The ‘elbow method’ was applied again to choose eight clusters as the point at which the variance in PSTH patterns explained by a model, which replaced each unit's PSTH with the average PSTH for the unit's cluster, began to level off with additional clusters (figure 6*c*). Of the eight clusters, one contained only 1 unit and was therefore excluded from further analysis, leaving a total seven clusters consisting of 248 single and multi-units.

### (c) Psychoacoustics

The experimental methodology was approved by the local Ethical Review Committee of the Experimental Psychology Department of the University of Oxford, and conforms to the ethical standards in the 1964 Convention of Helsinki.

Fifteen paid participants aged 22–45 with normal hearing were recruited. Three subjects were authors on this study, and five had >3 years of musical training. Subjects were instructed to listen to the rhythms that would emerge from a masking noise that was ramped down over 3 s, to begin tapping with a finger on a handheld button once they had found the beat, and to continue tapping steadily until the rhythm stopped. Stimuli were played through a TDT RM1 mobile processor (Tucker Davis Technologies, Alachue, FL, USA), and presented diotically at 50 dB SPL over Sennheiser HD 650 headphones (Wedemark, Germany). The TDT device delivered the stimuli and recorded button presses, allowing precise tap times to be measured. Patterns were randomly interleaved and presented a total of three times over the course of the experiment. Two subjects whose tapping patterns were not isochronous were excluded from further analysis, leaving a total of 13 subjects.

Consensus beat frequency and phase were determined for each stimulus based on the most common tapping pattern recorded from our human participants. ‘On-beat’ sounds were all sounds at a consensus tap location, and ‘off-beat’ sounds were all other sounds that did not coincide with a consensus tap location.

## Supplementary Material

Supplementary Materials (includes 4 figures)
