# Supplementary material for: Midbrain adaptation may set the stage for the perception of musical beat
Source: Proc Biol Sci. 2017 Nov 8;284(1866):20171455. doi: 10.1098/rspb.2017.1455 (PMC5698641; doi:10.1098/rspb.2017.1455)
Supplement: Supplementary Materials (includes 4 figures) [file rspb20171455supp1.docx]

# Supplementary Material


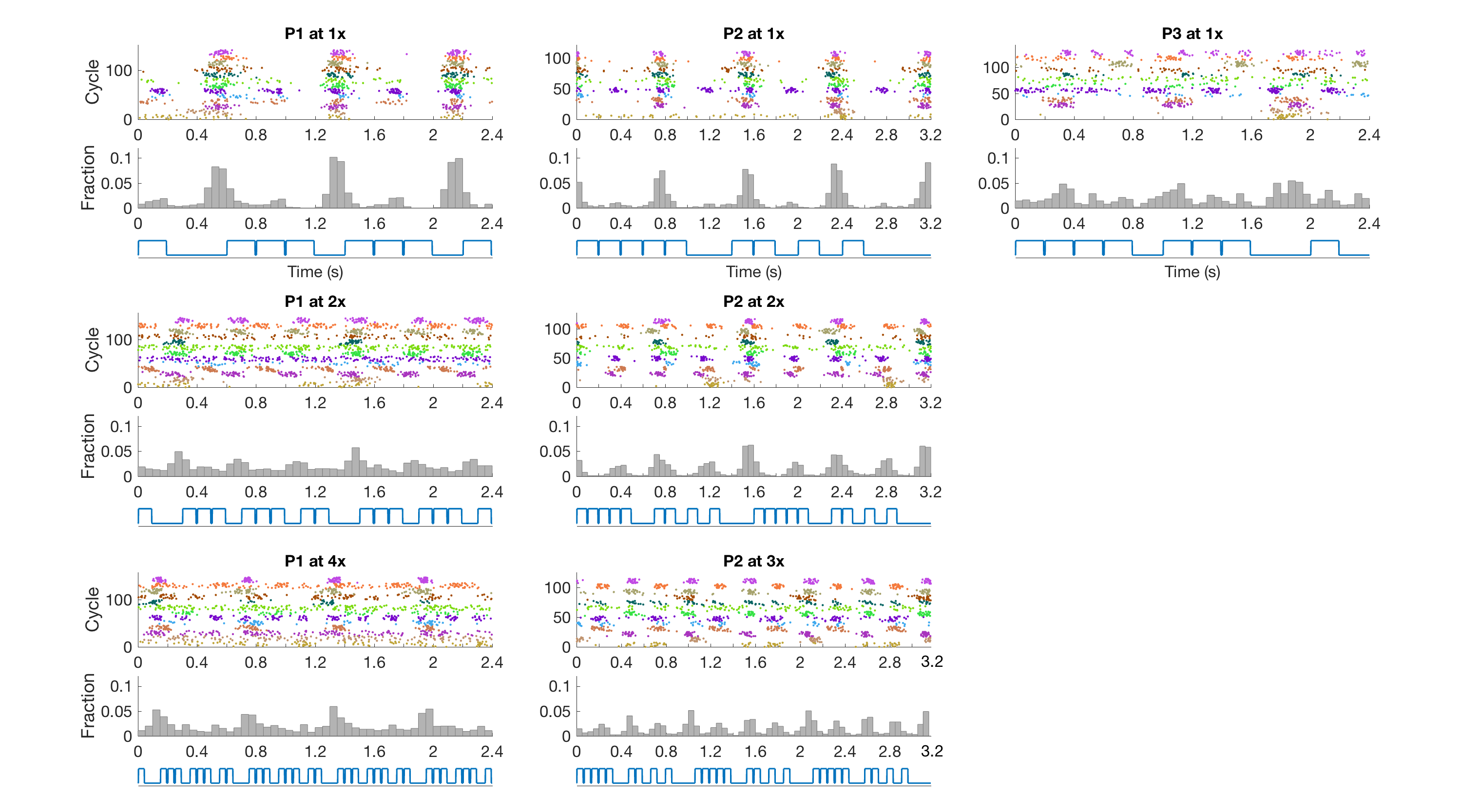


**Figure S1. Raster-style plot of tap times for each stimulus.** Each dot marks the timing of a tap, each row of dots shows tap responses for a single cycle of the stimulus pattern, and different colour dots distinguish the tap responses from different subjects. Below (in grey) is a histogram of tap times pooled across subjects, cycles, trials for each stimulus. At the bottom of each panel is the stimulus pattern.


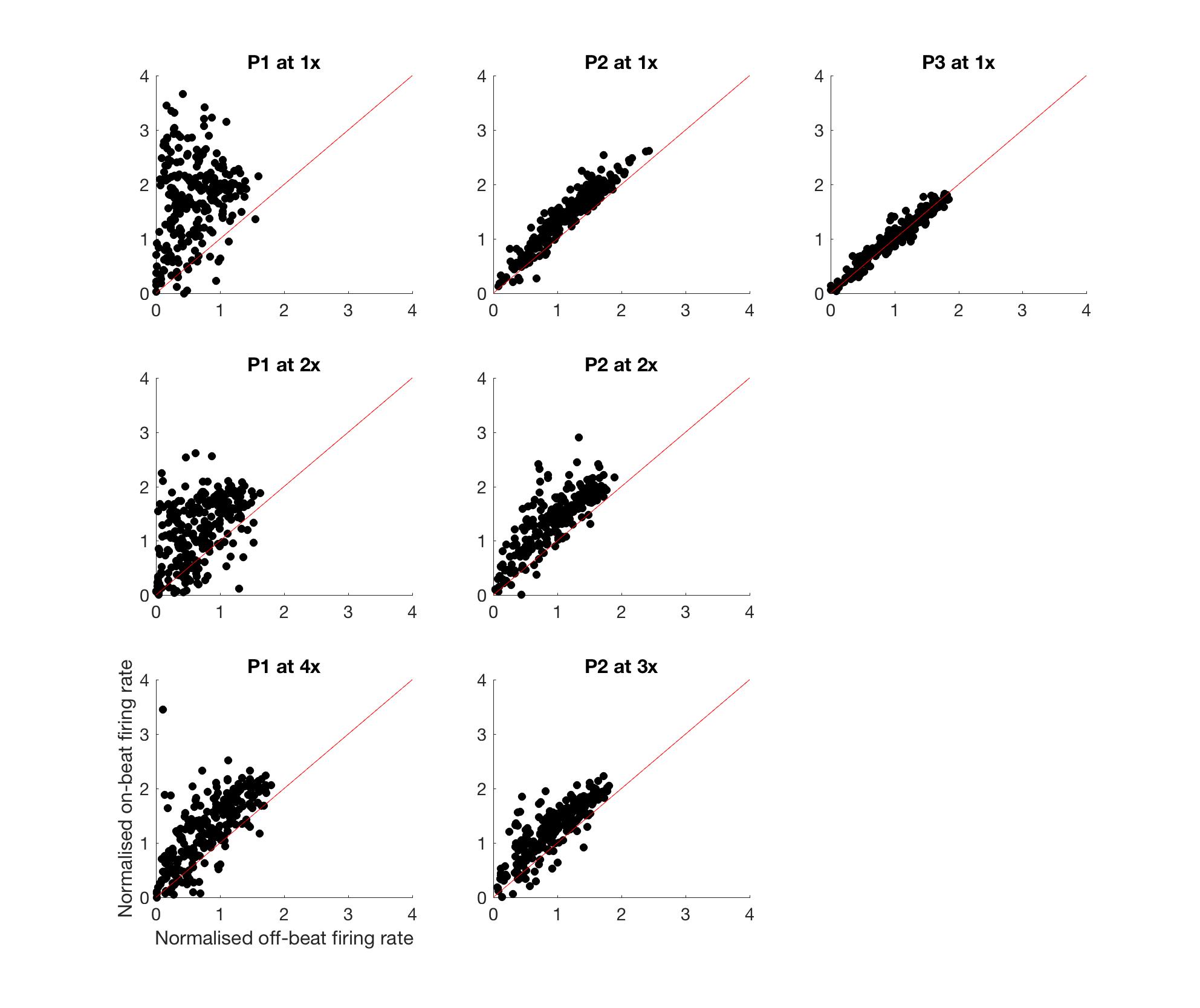


**Figure S2. On-beat firing rate versus off-beat firing rate for each stimulus.** All stimulus patterns except P3 show on-beat responses that are significantly larger than off-beat responses (p<0.0001, Wilcoxon paired signed rank test, Bonferroni corrected, N=248 units).


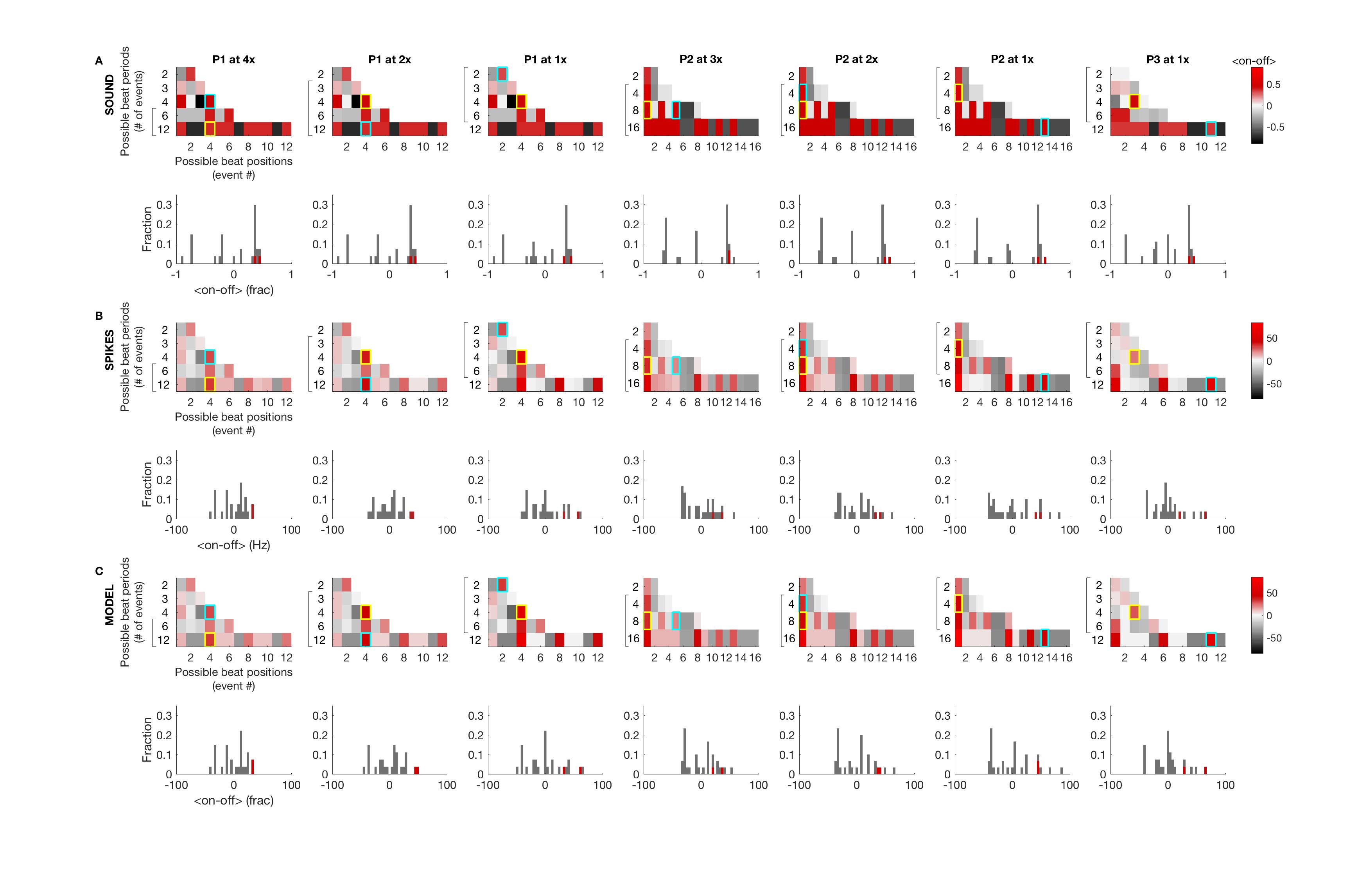


**Figure S3.** **Heatmaps of *on-off* values by stimulus.** **A)** Heatmaps of *on-off* values for sound for all plausible beat structures for each of the 7 stimuli. Yellow boxes mark the most commonly tapped beat structure, and cyan boxes mark the second most commonly perceived beat structure for each stimulus. The histograms directly below show the two most commonly perceived beat structures in red, and all possible beat structures in grey. Note that *on-off* values were calculated over each stimulus as played, which results in small discrepancies between the *on-off* histograms for sound for the same pattern presented at different rates. This is due to the looped pattern being cut off in slightly different places at the end of the 33 s. If the final incomplete loop of each stimulus pattern is cut off artificially, the sound *on-off* histograms for P1 at all rates and P2 at all rates become identical as expected.  **B)** Same as panel A, but for firing rates. **C)** Same as panel A, but for firing rates estimated by the exponential fits modelling adaptation. Note the close similarity between values in panel B and C.


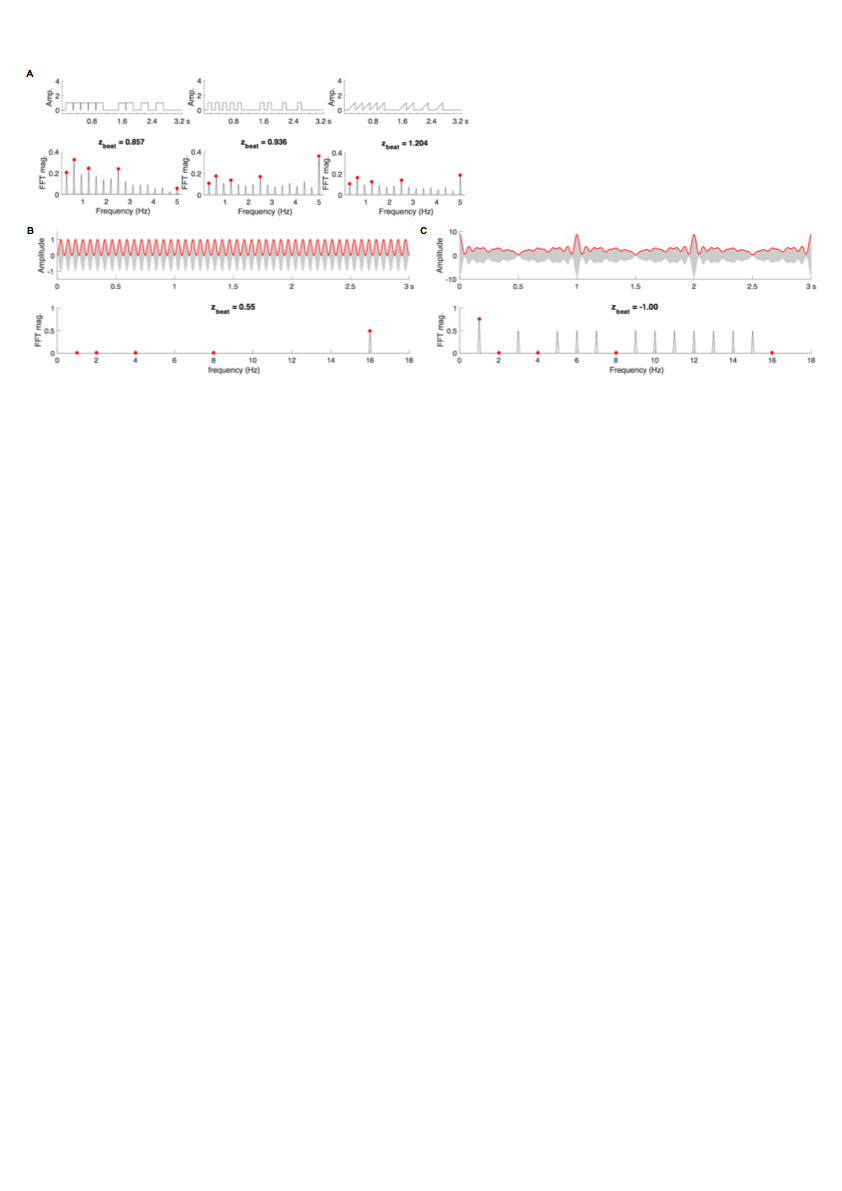


**Figure S4. Problems with the beat-related z-score metric used in Nozaradan, et al. (2012).**

Mean beat-related z-scores (referred to hereafter as MBRZs) were introduced in Nozaradan, et al. (2012) and used in a number of studies thereafter, e.g. [1,2] ^[[1]](#footnote-1)^, to quantify beat entrainment in either stimulus waveform envelopes or in neural response measures such as EEG waveforms. Unfortunately, MBRZs lack the specificity required to make them a reliable, unambiguous metric of beat entrainment.

This lack of specificity arises because MBRZs are calculated from Fourier amplitudes of a number of frequencies that are harmonics of the given or suspected underlying beat frequency. The rationale behind this seems reasonable at first, as the MBRZ asks whether the amplitudes of frequency components that are “beat related” are on average larger than the components that are not. However, the relative phases of these frequency components are not taken into account at all. This is a problem because harmonic frequency components with relatively modest amplitudes can interact to produce sizeable periodic transients in the time domain if all these components are in phase. Conversely, components with relatively large amplitudes may not produce much amplitude modulation at the common beat period if their phases do not favour constructive interference. By ignoring phase relationships, the MBRZ will miss potentially important periodic time domain features that can strongly affect the type of beat a signal may evoke when listened to. Additionally, changes in a signal’s waveform that have no bearing on the signal’s beat may change the MBRZ even if there is no obvious change in “beat strength”. These problems will be illustrated below.

To compute MBRZs, first the Fourier transform of the neural signal or the sound signal’s Hilbert envelope is calculated, and the amplitudes of the first 12 to 16 harmonics of the beat frequencies are determined. The amplitudes of these harmonics are then classified as “beat-related” or “beat-unrelated”. Beat-related frequencies are harmonics of the beat frequency that correspond to plausible beat groupings. Plausible beat groupings were 1, 2, 4, 8, 16 for 16-event patterns, and 1, 2, 4, 12 for 12-event patterns. Beat-unrelated frequencies are the harmonics that do not correspond to a plausible beat grouping, e.g. 3, 4, 7 and 9 - 15 for a 16-event pattern. The z-scores for each of these amplitudes is then computed, using z = (x-μ)/σ, where μ is the mean across both beat-related and beat-unrelated harmonics, and σ is their standard deviation. Finally, the average over the z-scores for the beat-related frequencies is calculated to yield the MBRZ.

In Nozaradan, et al. (2012), a larger MBRZ is taken as evidence for larger entrainment to beat or more beat-related activity, but as mentioned above, manipulations of a signal that have little to do with beat entrainment can also produce changes in the MBRZ. In Figure S4A, three variants of the sound envelope for pattern P2 at 1x tempo are shown. The rhythmic pattern remains the same throughout, but the envelope of the constituent sound events is either a wide rectangle (left), a narrower rectangle (middle), or a sawtooth (right). Below each waveform is the result of performing a Fourier transform on the corresponding envelope (only the low frequency end is shown, as higher frequencies do not enter into MBRZ calculations). Beat-related frequencies are marked by red circles. Note that the relative amplitudes of the beat-related and beat-unrelated frequencies change even though the rhythmic pattern remains the same. Consequently, there are also changes in the MBRZ values (shown above the spectra), but these changes to the MBRZs do not reflect anything to do with beat processing – an illustration of the lack of specificity of the MBRZ metric.

Panels B and C in Figure S4 illustrate the insensitivity of the MBRZ to important time domain features that arise from interactions between harmonics of the beat frequency. In Figure S4B, a signal envelope was created to have a positive MBRZ for a 1 Hz beat, even though it has no 1 Hz beat whatsoever. This was achieved simply by setting the amplitude of one of the highest beat-related frequencies to be large, and all other relevant harmonics of the beat frequency to be zero. In this case there can be no constructive interference to generate periodic features at the beat frequency. In Fig S4C, a signal envelope was created to have a negative MBRZ for a 1 Hz beat, but nevertheless has pronounced periodic transient features once every second, which, when listened to, produce a very salient 1 Hz beat. To create this envelope, we set the amplitude of the fundamental beat frequency to 0.75 but all other “beat related frequencies” to zero, and we set all non-beat related frequencies to have amplitude of 0.5. All frequency components are in cosine phase. Since most beat-related amplitudes are zero, they are on average smaller than the non-beat-related amplitudes, causing the MBRZ to be negative. But the fairly large single frequency component at the fundamental beat frequency interferes constructively with all the supposedly non-beat-related frequency components to produce periodic transients at the beat frequency. This evokes a very salient beat at 1 Hz despite the negative MBRZ. According to Nozaradan, et al. (2012), the difference in MBRZ should be evidence that the signal in B has “more” 1 Hz beat than the signal in C, but in these examples the opposite is the case. Because the MBRZ does not reliably measure what it is supposed to measure, applying MBRZ analysis to our data would not have resulted in a meaningful, interpretable comparison between our results and those of Nozaradan, et al. (2012). Below, we reproduce some Matlab code that interested readers can use to reproduce the example from Figure S4 themselves.

%% Sound demos: envelopes in Figure S4A

% 3 cycles of P2 at 1x = 9.6 s

fs = 44100;

noise = randn(1,9.6*fs);

%% SQUARE

s=[ones(1,.19*fs) zeros(1,.01*fs)]; % sound envelope

q=zeros(1,.2*fs); % silent gap

env=[s s s s s q q s s q s q s q q q]; % P2 PATTERN

env = repmat(env,1,3); % 3 cycles

square = env.*noise;

soundsc(square,fs)

%% SHORTER SQUARE

s=[ones(1,.1*fs) zeros(1,.1*fs)]; % sound envelope

env=[s s s s s q q s s q s q s q q q];

env = repmat(env,1,3);

shortsquare = env.*noise;

soundsc(shortsquare,fs);

%% SAWTOOTH

nsamp = .19*fs;

s=[linspace(0,1,nsamp) zeros(1,.01*fs)];

env=[s s s s s q q s s q s q s q q q];

env = repmat(env,1,3);

sawtooth = env.*noise;

soundsc(sawtooth,fs);

%% Make a signal without a beat at 1 Hz but a "false positive" MBRZ.

% as in Fig S4 B

beatinds= [1 2 4 8 16];

nonbeatinds = setdiff(1:max(beatinds),beatinds);

amps=zeros(1,16);

amps([16])=1;

zsnd = zscore(amps);

zbeat = mean(zsnd(beatinds))

znonbeat = mean(zsnd(nonbeatinds))

% make the actual envelope and show that it has no 1 s beat.

fs=20000;

step=2/fs;

theta=[0:step:(2-step)]*pi;

env=ones(size(theta));

for f=1:length(amps);

env=env+amps(f).*cos(f*theta);

end;

carrierF=1000;

carrier=sin(carrierF*theta);

asnd=carrier.*env;

figure(5); clf;

hold on;

t=(1 : 3*length(asnd))/fs;

plot(t,[asnd asnd asnd],'g');

plot(t,[env env env],'r','linewidth',2);

title(sprintf(' Zscore: %3.2f ',zbeat))

%% now let's listen: no beat at 1 Hz (it beats at 16)

outsnd=asnd;

outsnd=repmat(outsnd,1,6); % concat 6 fold

soundsc(outsnd,fs)

%% Make a signal with beat but a "false negative" MBRZ.

% as in Fig S4 C

beatinds= [1 2 4 8 16];

nonbeatinds = setdiff(1:max(beatinds),beatinds);

amps=zeros(1,16);

amps(nonbeatinds)=0.5;

amps([1])=0.75;

zsnd = zscore(amps);

zbeat = mean(zsnd(beatinds))

znonbeat = mean(zsnd(nonbeatinds))

% now make actual envelope of 1 s length

fs=20000;

step=2/fs;

theta=[0:step:(2-step)]*pi;

env=ones(size(theta))+10;

for f=1:length(amps);

env=env+amps(f).*cos(f*theta);

end;

carrierF=1000;

carrier=sin(carrierF*theta);

asnd=carrier.*env;

figure(5); clf;

hold on;

t=(1 : 3*length(asnd))/fs;

plot(t,[asnd asnd asnd],'g');

plot(t,[env env env],'r','linewidth',2);

% calculate MBRZ

zvals=zscore(amps);

MBRZ=mean(zvals(beatinds));

title(sprintf(' Zscore: %3.2f ',MBRZ))

%% now let's listen to the sound to make sure it really does beat at 1 Hz

outsnd=asnd;

outsnd=repmat(outsnd,1,6); % concat 6 fold

soundsc(outsnd,fs) % a clear 1 Hz beat, even though the z-score is negative

1. 1. Chemin, B., Mouraux, A. & Nozaradan, S. 2014 Body Movement Selectively Shapes the Neural Representation of Musical Rhythms. *Psychol Sci* **25**, 0956797614551161–2159. (doi:10.1177/0956797614551161)

   2. Nozaradan, S., Peretz, I. & Keller, P. E. 2016 Individual Differences in Rhythmic Cortical Entrainment Correlate with Predictive Behavior in Sensorimotor Synchronization. *Scientific Reports* **6**, 20612. (doi:10.1038/srep20612) [↑](#footnote-ref-1)
